# Key Components of Successful Management of Acute Fatty Liver of Pregnancy: A Case Report and Literature Review

**DOI:** 10.7759/cureus.53911

**Published:** 2024-02-09

**Authors:** Rekha Patidar, Krishi Gowdra Revannasiddappa, Muniza Ghazanfer

**Affiliations:** 1 Obstetrics and Gynecology, Zulekha Hospital, Dubai, ARE

**Keywords:** fetomaternal outcome, encephalopathy, liver diseases, coagulopathy, aflp

## Abstract

Acute fatty liver of pregnancy (AFLP) is a rare, potentially fatal obstetric emergency. Due to its nonspecific signs and symptoms, there is often a delay in diagnosis and management which is associated with morbid complications and high mortality. We report a case of a 30-year-old female gravida 3 para 2 at 32 weeks gestation who presented with nausea and vomiting for two weeks, pruritis for three days, and upper abdomen pain for a day. A clinical diagnosis of HELLP (hemolysis, elevated liver enzymes, and low platelets) syndrome/obstetric cholestasis/AFLP was made. Despite prompt management, her postpartum period was complicated by acute hepatic encephalopathy, hepatorenal shutdown, pancreatitis, coagulopathy, postpartum hemorrhage, and large abdominal wall hematoma. A high index of suspicion, prompt delivery, advanced critical support, and multidisciplinary team involvement led to successful fetomaternal outcomes in the patient.

## Introduction

Acute fatty liver of pregnancy (AFLP) is a rare, potentially fatal condition that occurs in the late second or third trimester or early postpartum period [1,2]. The condition is associated with a high mortality rate if not diagnosed and treated early. It was first described in the 1940s by Sheehan [3], as an “acute yellow atrophy of the liver”, as the histopathological picture typically shows microvesicular fatty infiltration of hepatocytes without inflammation or necrosis.

The incidence of AFLP is one in 7000 to one in 20,000 pregnancies [4,5]. It has a high maternal mortality rate ranging from 16.5% to 26.7% according to a recent study [6] and a neonatal mortality rate of 7-66% [7]. AFLP is a rare disorder that is a medical and obstetric emergency, which can lead to liver failure due to microvesicular fatty infiltration of hepatocytes [8]. Early diagnosis, prompt delivery, and supportive maternal care are important for favorable fetomaternal outcomes.

## Case presentation

A 30-year-old female, G3P2L1 with singleton gestation at 32 weeks, presented in the antenatal outpatient clinic with nausea, vomiting for two weeks, pruritus for three days, and upper abdominal pain for one day. On examination, the patient was conscious, oriented with a blood pressure of 130/90mmHg, and mildly dehydrated. Obstetric examination revealed a 30-week size uterus, non-tender, breech presentation. Ultrasound showed mild intrauterine growth restriction (IUGR).

The patient was admitted for observation with provisional diagnoses of pre-eclampsia, and intrahepatic cholestasis of pregnancy with mild IUGR. The workup was done given the above diagnoses which revealed elevated bilirubin, aminotransferases, and mildly elevated bile acid. Ultrasound whole abdomen, complete blood count, renal function test, and coagulation profile were in the normal range. The patient was commenced on intravenous fluids and antiemetic medication. Prophylactic antenatal corticosteroids were given. She improved symptomatically, and her blood pressure remained in the normal range during her hospital stay without medication, but the patient left against medical advice after 24 hours.

She was readmitted 12 hours after discharge with complaints of reduced fetal movements, labor pains, and show. On examination, she was well-oriented, and conscious but irritable in behavior, her vitals were stable, and obstetric examination revealed a 30-week uterus, breech presentation, with mild uterine contractions, cervical os closed, and mild show on vaginal examination. Cardiotocography (CTG) was non-reassuring with reduced variability. Repeat blood investigations revealed a raised white blood count, abnormal renal function test, abnormal liver function test, and deranged coagulation profile. However, platelet counts were in the normal range.

**Figure 1:**
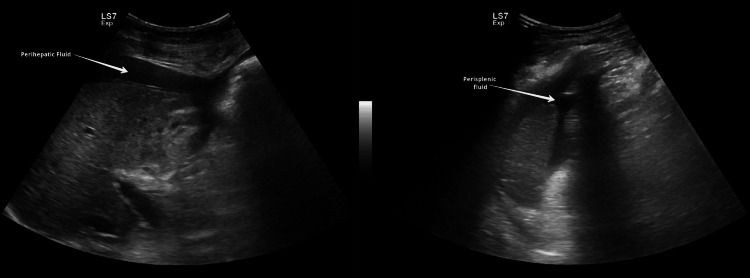
Ultrasound image shows perihepatic and perisplenic fluid suggestive of moderate ascites.

**Figure 2:**
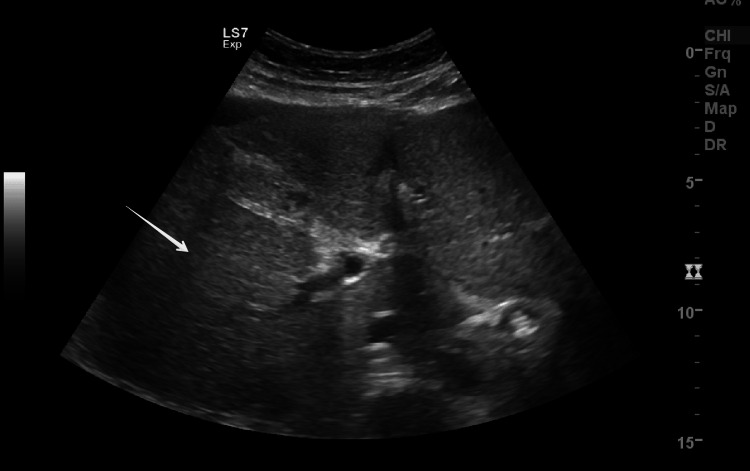
Ultrasound image shows increased echogenicity with fatty infiltration

**Figure 3:**
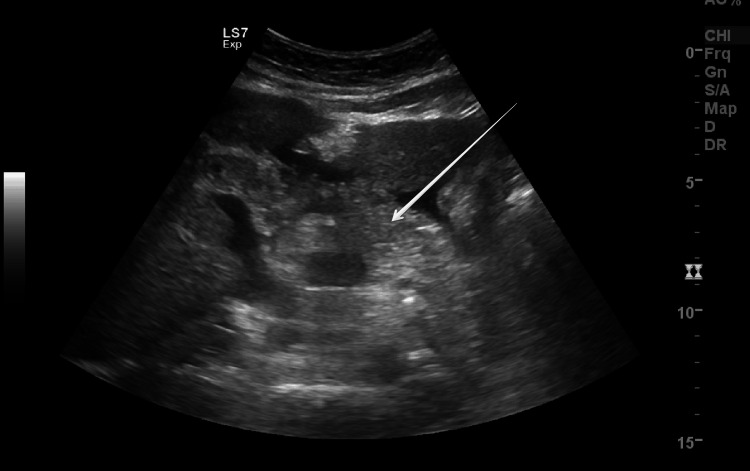
Ultrasound image shows pancreatic edema.

**Figure 4:**
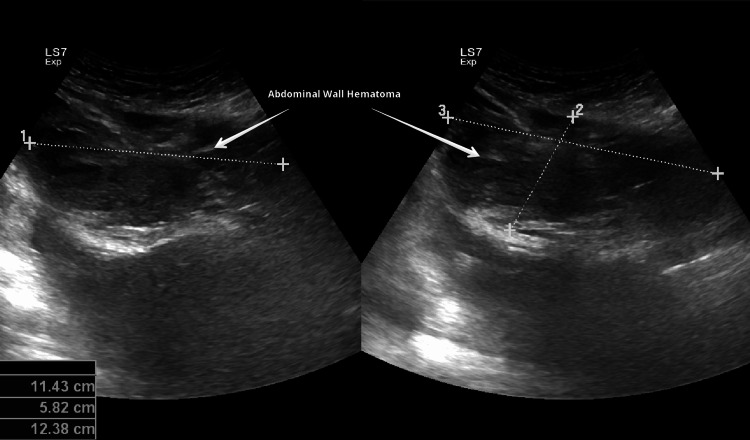
Ultrasound image shows midline infraumbilical hematoma of rectus sheath of size 11.4 cm x 5.8 cm x 12.3 cm.

Emergency cesarean section was done because of HELLP (hemolysis, elevated liver enzymes, and low platelets) syndrome/AFLP, non-reassuring CTG with breech presentation in labor. She delivered a live female baby weighing 1.4 kg with Apgar scores of 7, 8, and 9 at one, five, and 10 minutes respectively. There were no intraoperative complications. Postoperatively she was transferred to the intensive care unit (ICU) because of HELLP syndrome and AFLP for monitoring and care under a multidisciplinary team including obstetrician, intensivist, nephrologist, hematologist, gastroenterologist, and neonatologist. On the second postoperative day, her condition deteriorated, she became restless, and more irritable, had two episodes of postpartum hemorrhage (PPH), and developed abdominal distension due to ascites and progressive pedal edema. Her renal, liver, and coagulation profiles worsened further. Ultrasound revealed ascites (Figure 1), fatty changes in the liver (Figure 2), and pancreatic edema (Figure 3). PPH was managed by oxytocic medications and packed red blood cell transfusion. On the third postoperative day, she developed disseminated intravascular coagulopathy (DIC) and large midline infra umbilical hematoma of rectus sheath about 11.4 x 5.8 x 12.3 cm (Figure 4), altered sensorium, bilateral lower limb, and vulval edema. She was given supportive and medical management with the escalation of antibiotics, transfusion of packed red blood cells, fresh frozen plasma, and cryoprecipitate.

On the fifth postoperative day, she showed signs of clinical improvement and blood investigations. A conservative approach was adopted for abdominal wall hematoma. On the eighth postoperative day, she was transferred to the ward and discharged home on the 10th postoperative day in stable condition. Abdominal wall hematoma resolved by the fourth postoperative week. Complete resolution of laboratory investigations and ultrasound appearance took four weeks after delivery (Table 1).

**Table 1:** Laboratory investigations WBC: White blood cell; SGOT: Serum glutamic oxaloacetic transaminase; SGPT: Serum glutamic pyruvic transaminase; LDH: Lactate dehydrogenase; INR: International normalized ratio

Investigations	Day 0	Day 1	Day 2	Day 3	Day 4	Day 5	Day 10		Normal reference range
Hemoglobin, g/dl	12.4	11.1	8.7	4.4	6.6	8.8	9.8	12-15.5 g/dl	
WBC count X 10^3 ^/cu.mm	18.9	23.9	19	15.9	16.6	16	11.9	3.5-10.5 X 10^3 ^/cu.mm	
Platelets X 10^3^ /cu.mm	149	151	121	81	57	63	124	150-450 X 10^3 ^/cu.mm	
Total bilirubin, mg/dl	5.72	4.85	4.25	4.9	-	5.37	2.81	<1.1 mg/dl	
SGOT, U/L	74	57	51.5	47	49.2	64	39.6	<32 U/L	
SGPT, U/L	88	58	40.9	27	23.8	34	30.1	<33 U/L	
LDH, U/L	786		837	586	462	-	-	110-250 U/L	
Alkaline phosphate U/L	-	244	203	-	-	-	-	35-104 U/L	
Prothrombin time, sec	16.9	18.4	22.6	21.1	17.6	14.8	14.3	10.4-12.6 sec	
INR	1.43	1.55	1.89	1.77	1.49	1.26	1.21	0.8-1.2	
Activated prothrombin time, sec	39.4	37.9	46.6	38.8	29.4	26	32.7	24-36.2 sec	
Glucose, mg/dl	86	79	107	87	113	126	92	70-139 mg/dl	
Procalcitonin, ng/ml	-	1.38	-	6.4	-	2.65	-	< 0.049 ng/ml	
Amylase, U/L	-	32	-	-	-	-	-	28-100 U/L	
Fibrinogen, mg/dl	-	-	78	135	145	145	291	220-496 mg/dl	
Urea, mg/dl	69.9	76	96.5	132	134.5	107.8	23.3	< 50 mg/dl	
Creatinine, mg/dl	2.37	2.3	2.1	2	1.67	1.2	0.54	0.6-1.1 mg/dl	

The neonate was kept in the neonatal intensive care unit (NICU) because of preterm delivery and IUGR; however, he needed minimal supportive care and was discharged two weeks later. Genetic testing for long-chain 3-hydroxyacyl-CoA dehydrogenase (LCHAD) mutation was offered but the parents refused. 

## Discussion

AFLP is one of the serious liver disorders unique to pregnancy, diagnosis of which is often delayed due to nonspecific symptoms. The disease course is usually rapid and unpredictable and has the potential to develop life-threatening complications.

Risk factors

Risk factors include nulliparity, advanced maternal age, male fetus, previous history of AFLP, multiple gestations, low body mass index less than 20 kg/meter square, and co-existing liver disease [8,9]. In some cases, as in the current case, AFLP occurs even in the absence of the above-mentioned risk factors, stressing the importance of a high index of suspicion in patients presenting with nonspecific symptoms for early diagnosis of AFLP.

Etiology

Though the precise etiology of AFLP is unclear, the possible reason for AFLP may be mitochondrial dysfunction and abnormal fatty acid metabolism in pregnancy. The process of mitochondrial fatty acid beta-oxidation consists of a series of transport steps and four enzymatic reactions [1]. This pathway generates energy from free fatty acids for the brain, heart, liver, and skeletal muscles during fasting when the glycogen stores are depleted. Deficiency of the third enzyme, LCHAD results in the accumulation of medium and long-chain fatty acids in maternal blood and hepatocytes, with toxic effects on maternal hepatocytes. It is an autosomal recessive disorder and heterozygous LCHAD deficiency has been identified in some women with AFLP [9,10]. Approximately 20% of AFLP is associated with LCHAD deficiency [11] The homozygous G1528C mutation, which alters amino acid 474 from glutamic acid to glutamine on protein (E474Q), appears to be the most common genotype associated with the development of AFLP [12]. It was hypothesized that in fetuses homozygous for LCHAD deficiency, the fetus will be unable to oxidize long-chain fatty acids. The unmetabolized free fatty acids return via the placenta to the mother’s circulation, which strains maternal hepatic activity and overwhelms diminished maternal hepatic enzyme activity resulting in the accumulation of medium and long-chain fatty acids in maternal blood and hepatocytes resulting in toxic effects and the symptoms of AFLP [1].

Clinical presentation

Patients often present with nonspecific symptoms such as anorexia, nausea, vomiting, malaise, fatigue, headache, and abdominal pain. On physical examination, common signs are fever, jaundice, and tenderness in the right upper quadrant or mid-epigastric area. Concurrent preeclampsia is noted in 20-40% with 20% also being diagnosed with HELLP syndrome [13]. In severe cases, the disease progresses rapidly resulting in signs and symptoms of acute liver failure, encephalopathy, hypoglycemia, acute renal failure, disseminated intravascular coagulopathy, gastrointestinal bleeding, and pancreatitis, and often progresses to multiorgan failure. Diminished uteroplacental perfusion may occur due to maternal hypovolemia and metabolic acidosis which leads to harmful effects on fetal well-being [14].

Differential diagnoses

AFLP is a diagnosis of exclusion. The differential diagnoses are mainly HELLP syndrome and pre-eclampsia with abnormal liver enzymes. Not only is a large clinical overlap present between AFLP, HELLP, and severe preeclampsia, but these may also coexist. Concurrent preeclampsia is seen in 70% of women with AFLP. Hypertension is present in 100% of patients with pre-eclampsia, 85% of patients with HELLP, and about 50% of patients with AFLP. Severe symptoms and signs of hepatic insufficiency like hypoglycemia, ascites, encephalopathy, coagulopathy, acute liver failure, and multiorgan involvement are more common in AFLP [15]. Severe hypertension and proteinuria are more consistent with HELLP and severe preeclampsia. The distinctive histology of HELLP syndrome is the presence of prominent periportal hemorrhages and fibrin deposition, whereas micro-vesicular fatty infiltrations are characteristic of AFLP.

In addition, intrahepatic cholestasis of pregnancy, and non-pregnancy-related conditions causing abnormal liver enzymes, such as acute viral hepatitis, autoimmune hepatitis, drug-induced hepatitis, gallstones, gastroenteritis, and Budd-Chiary syndrome should be explored [1,6]. Detailed history, examination, and laboratory investigations help in differentiating AFLP from other liver diseases in pregnancy (Table 2).

**Table 2:** Characteristics of common liver diseases in pregnancy ALP: Alkaline phosphatase; ALT: Alanine aminotransferase; DIC: Disseminated intravascular coagulation; GGT: Gammaglutamyl transpeptidase; HELLP: Hemolysis, elevated liver enzymes, low platelets; IUGR: Intrauterine growth restrictions; LCHAD: Long-chain 3-hydroxyacyl-CoA dehydrogenase; LDH: Lactate dehydrogenase; PT: Prothrombin time Table source: Ko and Yoshida, 2006 [1]; Copyright: 2006 Hindawi Publishing Corporation, published under creative commons license

Disease	Trimester	Incidence	Signs and symptoms	Laboratory findings	Complication
Pre-eclampsia and Eclampsia	2^nd^ or 3^rd^	5-10%	Nausea, vomiting, epigastric pain, edema, hypertension, altered mental state, jaundice (late presentation)	ALT <500U/L, proteinuria, DIC (7%)	Maternal: Hypertensive crisis; renal impairment; hepatic rupture/infarct; neurological (seizures, cerebrovascular disease)
					Fetal: Abruptio placentae; prematurity; IUGR leading to increased perinatal morbidity and mortality
HELLP Syndrome	3^rd^	0.10% (4-12% in case of pre-eclampsia)	Symptoms of pre-eclampsia (hypertension, headache, blurred vision); epigastric or right upper quadrant pain; nausea; vomiting; hematuria; jaundice (late presentation	Hemolysis, ALT <500 U/L, platelets <100×10^9^/L, elevated LDH, DIC (20%–40%)	Maternal: Seizures; acute renal failure; hepatic rupture, hematoma or infarct; increased mortality (1-3%)
					Fetal: Abruptio placentae; increased mortality (35%)
Acute Fatty Liver of Pregnancy	Late 2^nd^ or 3^rd^ trimester	0.01%	Malaise, upper abdominal pain, nausea, vomiting, jaundice (very common), encephalopathy (late presentation )	ALT <500 U/L; hyperbilirubinemia; hypoglycaemia; elevated ammonia; leucocytosis; DIC (>75%) – thrombocytopenia, prolonged PT, hypofibrinogenemia	Maternal: Acute renal failure; encephalopathy; ascites; sepsis; wound seroma; pancreatitis; increased mortality
					Fetal: Increased mortality (13% to 18%) from asphyxia; prematurity; IUGR; LCHAD deficiency and its complications
Viral hepatitis	Any	Same as general population	Nausea, vomiting, fever	ALT greatly elevated (>500 U/L); elevated bilirubin; positive serology tests	Increased mortality with hepatitis E
Intrahepatic cholestasis of pregnancy	2^nd^ or 3^rd^	0.1-0.2%	Intense pruritus; jaundice; (20-60%, 1-4 weeks after pruritus); steatorrhea	ALT <500 U/L; markedly elevated ALP and GGT; increased bile acids; bilirubin (<103 μmol/L)	Maternal: Predisposed to cholestasis in subsequent pregnancies.
					Fetal: Stillbirth; prematurity; fetal mortality (3.5%)
Drug-induced hepatitis	Any	Unknown	Usually none; nausea; vomiting; pruritis; jaundice (in cholestatic hepatitis)	Variable	Unknown

Diagnosis and diagnostic criteria

Reyes reported a large number of AFLP cases could survive if they were delivered within a week after the onset of the disease and that 30% would die if they were delivered beyond two weeks after the onset of the disease [7]. To lower the mortality, early diagnosis is important. The diagnosis of AFLP remains mainly clinical and the Swansea criterion is commonly used to diagnose AFLP (Table 3). The criteria include symptoms, signs, biochemical and imaging findings, where the presence of six or more of its components, in the absence of another cause, helps in establishing a clinical diagnosis of AFLP [9,12]. Swansea criteria were applied to a cohort of 24 patients with suspected pregnancy-related liver disease who underwent biopsy, and the presence of six or more components had a positive predictive value of 85% and a negative predictive value of 100% for finding micro-vesicular steatosis [16].

**Table 3:** Swansea criteria for diagnosis of acute fatty liver of pregnancy The presence of six or more features in the absence of another cause helps in diagnosis of acute fatty liver disease of pregnancy Table adapted from: Naothavorn et al., 2022 [9]; Published under Creative Common Attribution - NonCommercial- NoDerivatives 4.0 International

Criteria	Features
Clinical symptoms	Vomiting
	Abdominal pain
	Polydipsia/Polyuria
	Encephalopathy
Laboratory parameters	Elevated bilirubin (>0.8mg/dl)
	Hypoglycaemia (<72mg/dl)
	Elevated uric acid (>5.7mg/dl)
	Leucocytosis (>11 x 10^9^L)
	Elevated transaminases (>42U/L)
	Elevated creatinine (>1.7md/dl)
	Elevated ammonia (>66ug/dl)
	Coagulopathy (Prothrombin time >14 sec)
Imaging	Ascites or bright appearing liver on ultrasound
Histology	Microvesicular steatosis on liver biopsy

In the current case, nausea, vomiting, abdominal pain, and later encephalopathy, jaundice, raised bilirubin, liver enzymes, coagulopathy, and mild thrombocytopenia were present, although hypoglycemia or raised amylase/lipase was not present.

Management

Early diagnosis, prompt delivery, and intensive supportive care are the cornerstones in the management of AFLP as it decreases mortality from 85% [17] to 12.5% [4,7]. Although there is no consensus on the best method of delivery, most guidelines recommend prompt and safe delivery. Induction of labor and vaginal delivery was associated with a decreased risk of maternal and fetal mortality as reported by Burroughs et al. in 1982 [14]. The risk of intraabdominal bleeding is higher in cases of cesarean section. Recent literature reports cesarean delivery in 65% of cases [4,18]. This may be a reflection of women presenting with more severe disease needing to expedite delivery. According to the best of our knowledge, there have been no randomized control trials comparing vaginal and cesarean delivery in AFLP patients. Current evidence favors cesarean section even though the risk of intraabdominal bleeding is high. A meta-analysis by Wangt al. involving 1350 subjects published in 2016 showed cesarean section had a positive effect on two of the three primary outcomes. The maternal mortality rate was 44% lower (RR: 0.56) and the perinatal mortality rate was also reduced (RR: 0.52). No significant difference was found in terms of liver failure-associated complications (hypoglycemia, ascites, encephalopathy, DIC, other organ injuries, obstetric hemorrhage, and infection). If vaginal delivery is preferred, vaginal trauma such as episiotomy should be avoided [18]. If a cesarean section is planned, then a vertical midline skin incision is preferred due to the natural avascular surgical plane [18]. Pfannenstiel incision is more commonly associated with hematoma at the level of rectus sheath in AFLP patients with concurrent coagulopathy. Surgical drains, intraperitoneal and subrectal may be considered.

If AFLP is complicated with PPH, intrauterine balloon tamponade has a known beneficial effect in controlling PPH even with liver failure and deranged coagulation profile [19]. Delivery plans should be individualized for each patient as many factors contribute to decision making including severity of disease, coexisting conditions, fetal condition, laboratory values, maternal obstetric history, and provider experience. Care should be taken to avoid massive blood loss attributable to coagulopathy by timely transfusion of fluids and blood products. Fresh frozen plasma or cryoprecipitate should be transfused with a goal fibrinogen level of at least 150 mg/dl and platelet transfusion to keep platelet count above 50,000/ ml. Some studies have indicated the role of pre/intra/postoperative use of recombinant factor seven A (rFVII a) in pregnant patients with liver failure and coagulopathy with massive bleeding being taken up for emergent surgery [20]. This may decrease the number of blood products administered and avoid hysterectomy. Patients are also at risk of hypoglycemia and may need monitoring and intravenous glucose administration. Other potential complications of AFLP such as pancreatitis, risk of rupture, and renal dysfunction should be diagnosed early and managed. Most patients experience clinical recovery within three to four days after delivery and complete recovery in 7-10 days. Patients frequently have initial worsening of clinical condition, renal function, liver function, and coagulopathy in the immediate postpartum period and require continued monitoring. Patients who develop multiorgan failure require advanced critical and supportive management which includes mechanical ventilation, dialysis for acute renal failure, nutritional support, and transfusion of blood products for coagulopathy and hemorrhage. Patients not improving with supportive care may need plasma exchange or liver transplantation. Increased risk of perinatal morbidity and mortality is due to fetal hypoxia secondary to maternal acidosis and preterm birth. Neonates, in whom LCHAD deficiency is detected, need long-term monitoring. Multidisciplinary team care involving an obstetrician, anesthetist, intensivist, hepatologist, hematologist, neonatologist, nephrologist, pathologist, blood bank, and advanced critical care support is of utmost importance for the safe and appropriate management of the patient.

Recurrence

The exact risk of recurrence is unknown. AFLP has been reported in subsequent pregnancies in patients who were found negative for LCHAD deficiency mutation.

## Conclusions

As most patients present with nonspecific symptoms, a high index of suspicion is crucial in patients with a new onset of nausea and vomiting in the third trimester and should be evaluated for AFLP. Prompt delivery is the definitive treatment as the progress of the disease can be rapid and unpredictable. Obstetric management strategies including timing and method of delivery require consideration of individual patients, coexisting conditions, and the anticipated clinical course of disease. Postpartum recovery may be prolonged and complicated due to potentially fatal complications that require a multidisciplinary approach coupled with advanced critical care support for favorable fetomaternal outcome.

## References

[1] Ko H, Yoshida EM (2006). Acute fatty liver of pregnancy. Can J Gastroenterol.

[2] Zhong Y, Zhu F, Ding Y (2020). Early diagnostic test for acute fatty liver of pregnancy: a retrospective case control study. BMC Pregnancy Childbirth.

[3] Sheehan HL (1940). The pathology of acute yellow atrophy and delayed chloroform poisoning. J Obstet Gynaecol Br Emp.

[4] Knight M, Nelson-Piercy C, Kurinczuk JJ, Spark P, Brocklehurst P (2008). A prospective national study of acute fatty liver of pregnancy in the UK. Gut.

[5] Allen AM, Kim WR, Larson JJ, Rosedahl JK, Yawn BP, McKeon K, Hay JE (2016). The epidemiology of liver diseases unique to pregnancy in a US community: a population-based study. Clin Gastroenterol Hepatol.

[6] Gao Q, Qu X, Chen X, Zhang J, Liu F, Tian S, Wang C (2018). Outcomes and risk factors of patients with acute fatty liver of pregnancy: a multicentre retrospective study. Singapore Med J.

[7] Rajasri AG, Srestha R, Mitchell J (2007). Acute fatty liver of pregnancy (AFLP)--an overview. J Obstet Gynaecol.

[8] Lim E, Mouyis M, MacKillop L (2021). Liver diseases in pregnancy. Clin Med (Lond).

[9] Naothavorn W, Thanapongpibul C, Sriudomporn K, Ruangkit C, Srivanitchapoom N, Tungtrongchitr N (2022). A 24-year-old woman presenting in the third trimester of pregnancy with nausea, vomiting, and abdominal pain and diagnosed with acute fatty liver of pregnancy. Am J Case Rep.

[10] Wanders RJ, Vreken P, den Boer ME, Wijburg FA, van Gennip AH, IJlst L (1999). Disorders of mitochondrial fatty acyl-CoA beta-oxidation. J Inherit Metab Dis.

[11] Tran TT, Ahn J, Reau NS (2016). ACG clinical guideline: liver disease and pregnancy. Am J Gastroenterol.

[12] Yang Z, Yamada J, Zhao Y, Strauss AW, Ibdah JA (2002). Prospective screening for pediatric mitochondrial trifunctional protein defects in pregnancies complicated by liver disease. JAMA.

[13] Liu J, Ghaziani TT, Wolf JL (2017). Acute fatty liver disease of pregnancy: updates in pathogenesis, diagnosis, and management. Am J Gastroenterol.

[14] Burroughs AK, Seong NH, Dojcinov DM, Scheuer PJ, Sherlock SV (1982). Idiopathic acute fatty liver of pregnancy in 12 patients. Q J Med.

[15] Casey LC, Fontana RJ, Aday A (2020). Acute liver failure (ALF) in pregnancy: how much is pregnancy related?. Hepatology.

[16] Goel A, Ramakrishna B, Zachariah U, Ramachandran J, Eapen CE, Kurian G, Chandy G (2011). How accurate are the Swansea criteria to diagnose acute fatty liver of pregnancy in predicting hepatic microvesicular steatosis?. Gut.

[17] Kaplan MM (1985). Acute fatty liver of pregnancy. N Engl J Med.

[18] Nelson DB, Byrne JJ, Cunningham FG (2020). Acute fatty liver of pregnancy. Clin Obstet Gynecol.

[19] Thakur N, Kishore R, Tuwani M (2021). Sooner than later: a little effort may avert postpartum hemorrhage in patients with acute hepatitis E. Int J Reprod Contracept Obstet Gynecol.

[20] Singh S, Menon A, Thareja S (2011). Recombinant activated factor VIIa in a case of pregnancy with acute hepatic failure and massive blood loss. Med J Armed Forces India.

